# Exploring the implications of case selection methods for psychiatric molecular genetic studies

**DOI:** 10.1038/s41380-025-03015-y

**Published:** 2025-04-20

**Authors:** Kenneth S. Kendler, Henrik Ohlsson, Jan Sundquist, Kristina Sundquist

**Affiliations:** 1 Virginia Institute for Psychiatric and Behavioral Genetics, Virginia Commonwealth University, Richmond, VA USA; 2 Department of Psychiatry, Virginia Commonwealth University, Richmond, VA USA; 3 Center for Primary Health Care Research, Department of Clinical Sciences, Lund University, Malmö, Sweden; 4 University Clinic Primary Care Skåne, Region Skåne, Sweden

**Keywords:** Genetics, Diagnostic markers

## Abstract

Researchers selecting probands for molecular genetic studies confront a range of sampling issues with modest empirical guidance. In this paper, using cases of major depression (MD), anxiety disorders (AD) alcohol use disorder (AUD), drug use disorder (DUD), bipolar disorder (BD) and schizophrenia (SZ) from a large population cohort of all native Swedes born 1940–2003, we examine the implications of three proband selection decisions by exploring profiles of genetic risks assessed using the validated family genetic risk scores. The impact of censoring cases with comorbid diagnoses is quite variable, depending on the frequency of that disorder in the case sample and the genetic relationship of the censored to the primary disorder. In an MD cohort, censoring SZ cases produces only a focal small decrease in schizophrenia genetic risk while censoring AD cases produces a wide-spread reduction in genetic risk for MD and most other disorders. We examine the value of censoring cases of SZ, BD and MD whose onset was preceded by one to two years by first episodes of DUD or AUD. We do not see any increase in genetic risk for these “screened” cohorts. Secondary ascertainment, where disorder A is ascertained as a comorbid diagnosis in a sample collected for disorder B, can, in certain situations, produces large increases in the genetic risk for disorder B and associated disorders in cases of A. However, if disorder B is closely genetically related to disorder A (as seen with MD/AD and DUD/AUD pairings), the pattern differs dramatically and produces a general moderate elevation across the genetic risk profile. These findings provide guidelines for future investigators and suggest caution when screening out comorbid disorders and when utilizing secondary ascertainment.

## Introduction

The current leading paradigm in psychiatric and substance use disorder genetics is the case-control genome wide association study (GWAS) that localizes common variants genome that differ significantly in their frequency in matched cases and controls. While some simulations have addressed the optimal sampling approach for controls in such studies and potential problems there-in [1, 2], less attention has focused on the selection of cases.

In this paper, we address possible biases that might arise from three common issues in the sampling of GWAS cases by calculating family -genetic risk scores (FGRS) from the nationwide Swedish population and health registers. The first, the *general comorbidity problem*, focuses on lifetime comorbidity. Comorbidity among psychiatric and drug use disorders is widespread [3–8], and would be expected in cases ascertained for genetic analysis. At least three possible approaches are available to an investigator to study disorder A in a cohort which also contains diagnostic information on comorbid disorders B, C, D …. One could study only cases of disorder A with no other psychiatric disorder or disorder A in “its pure form.” An alternative would be to exclude particular comorbid disorders considered to be especially problematic – e.g., cases with comorbid schizophrenia from a study of major depression (MD). The third approach would be to ignore comorbidity and study all cases of disorder A as they come.

The second question – which we call the *special comorbidity problem* -- is a particular case of the general comorbidity problem but focuses on specific prevalence windows and arises because of the concern that disorder X might predispose to the development of disorder Y. For example, psychotic symptoms sometime occur during episodes of substance use disorders some cases of which go on to develop a schizophrenia-like clinical picture and course [9–12]. Would it be wise for a schizophrenia genetics researcher to exclude all cases of schizophrenia that had episodes of DUD with a year or two prior to the disorder onset? Might that approach, if well empirically supported, eliminate some subjects whose schizophrenia arose largely from their exposure to psychoactive drugs of abuse rather than their genetic risk?

The third question involves the wisdom of *secondary ascertainment*. It arises when an investigator is forming a study of cases of disorder X but find few cohorts where disorder X is primarily ascertained. However, a number of cohorts of cases of disorders A, B and C exist where disorder X was evaluated as a comorbid disorder. Including such secondarily ascertained cases will increase the sample size of the disorder X cohort and provide more power. Examples of such secondary ascertainment are several GWAS studies of opiate use disorder (OUD) which included subjects primarily ascertained for alcohol use disorder (AUD) but who had a comorbid diagnosis of OUD [13, 14] and a major GWAS study of anxiety disorders which included cases ascertained for MD but who also had comorbid anxiety syndromes [15]. What kind of heterogeneity or bias might be introduced by utilizing such secondarily ascertained cases?

Instead of a simulation study designed to address these questions, we decided to examine empirical data based on our FGRS using extensive Swedish registry data (e.g., [16–20]). We selected seven psychiatric and substance use disorders – MD, Anxiety Disorders (AD), OCD, alcohol use disorder (AUD), DUD, BD and SZ—and examined these three questions in the nationwide data from Sweden. We focus on genetic risks not just for the primary disorder whose ascertainment is being altered but also the profile of a range of psychiatric and substance use disorders. This permits us to provide a broader view of the impact of ascertainment changes. Furthermore, we needed a metric which would index the change in genetic risk for each of these disorders in the profile induced by changes in the ascertainment strategy for the probands. We utilized an *FGRS Ratio*, explained in more detail below, which is defined as the ratio of genetic risk for a particular disorder A (e.g., AUD) in a representative cohort of cases of disorder B (e.g., MD) where a particular psychiatric disorder C (e.g., DUD) has been screened out compared to the level of genetic risk for disorder A (AUD) in an unselected cohort of cases of disorder B (MD).

## Methods

We collected information on individuals from Swedish population-based registers with national coverage linking each person’s unique personal identification number which, to preserve confidentiality, was replaced with a serial number by Statistics Sweden. We secured ethical approval for this study from the Regional Ethical Review Board in Lund and no participant consent was required (No. 2008/409 and later amendments). Our database consisted of all individuals born in Sweden from (1940 to 2003) to Swedish born parents (N = 5,828,049). In the database, we included age of first registration for the seven disorders MD, AD, OCD, AUD, DUD, BD and SZ, utilizing ICD-8, 9 and 10 codes from primary care, specialist, and hospital registers as well as prescription and criminal registries (see Appendix Tables 1–2). We also implement a hierarchy for the diagnoses of BD and SZ to assign a single diagnosis as outlined in Appendix Table 3. In the database, individual Familial Genetic Risk Scores (FGRSs) for the seven disorders were also included. Similar to prior studies, the FGRS were based on 1st–5th degree relatives to the probands with a mean of 40.1 relatives per proband. Briefly (see Appendix Table 4), they are calculated from morbidity risks for disorders in relatives, controlling for cohabitation, and thus arise from phenotypes in extended pedigrees, not from molecular genetic data. For further details and relevant simulations see [17]. They are standardized by year of birth and county of residence into a z-score with mean = 0 and s.d. = 1.

**Fig. 1:**
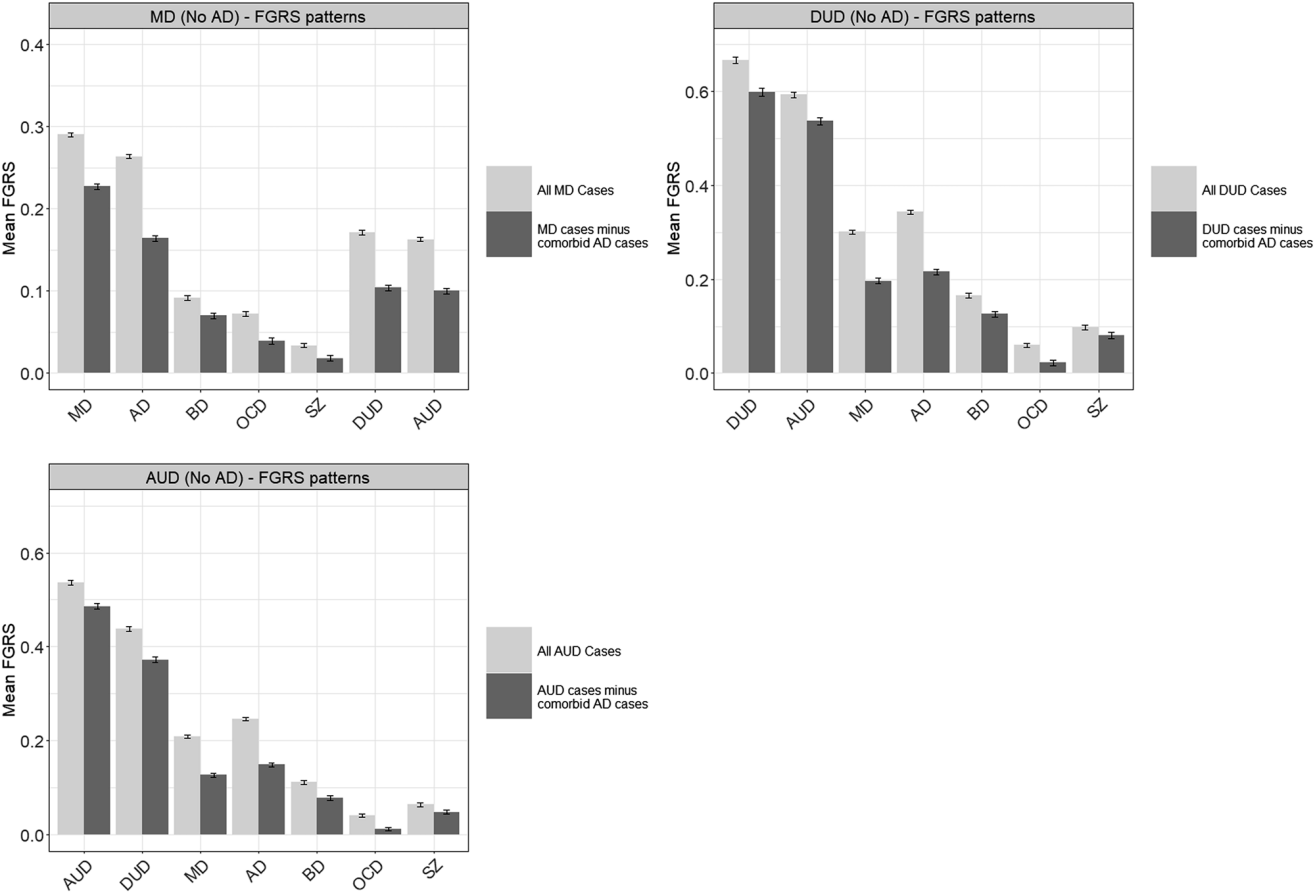
An example of results of the family genetic risk score (FGRS) (with 95% CIs) pattern of results in cases of major depression (MD), drug use disorder (DUD) and alcohol use disorder (AUD) with and without cases with comorbid anxiety disorders (AD). The Y-Axis is the mean FGRS (calculated as a standardized z score) for all MD cases in light grey and MD cases minus those cases with comorbid Anxiety Disorder (AD) in dark grey. The other initials in the figure as BD bipolar disorder, OCD obsessive-compulsive disorder, and SZ schizophrenia.

We calculated FGRS correlations by dividing each FGRS into 4 groups, identified via K-means clustering (see Appendix Table 5 and Fig. 1 for further details), and then obtaining polychoric correlations (see Appendix Table 6 for details). This statistic is not comparable to polygenic correlations because polygenic risk scores, based on thousands of individual variants, are normally distributed [21, 22]. FGRS scores, typically based on 30–50 relatives per pedigree have, especially with rare disorders, highly skewed distributions. Therefore, while values for FGRS correlations are generally proportional to those found for polygenic risk scores, they are all moderately to substantially lower.

To investigate general comorbidity, we adopted the simple statistic of a FGRS ratio. For example, in trying to quantify the change in genetic profile of cases of MD when all cases of comorbid AD are screened out, we calculate the FGRS ratio for the FGRSs for MD, AD, BD, OCD, SZ, DUD and AUD. In the calculations the denominator was the FGRS of cases of MD without any registrations for AD, and the numerator was the FGRS for cases of MD regardless of the occurrence of AD registrations. A FGRS ratio of 0.78, illustrates that the estimate of the FGRS_MD_ in MD cases with all AD comorbid cases removed is 78% that of the level of FRGS_MD_ found in unscreened MD cases. In the analyses we focus on cases of MD, DUD, AD and AUD, and we quantify the changes in the FGRSs for MD, AD, BD, OCD, SZ, DUD and AUD.

To investigate special comorbidity, we investigate the mean genetic risks for MD, BD and SZ cases (disorder A) who did versus did not have prior onsets of AUD and DUD (disorder B). We used three separate time periods to eliminate cases; registration of disorder B at any time prior to onset of disorder A, registration of disorder B within a 2-year period prior to onset of disorder A, and registration of disorder B within a 1-year period prior to onset of disorder A.

To investigate secondary ascertainment, we used the FGRS ratio. For example, in trying to quantify the change in genetic profile of cases of MD collected for AD, we calculate the FGRS ratio for the FGRSs for MD, AD, BD, OCD, SZ, DUD and AUD. In the calculations the denominator was the FGRS of cases of MD from a sample collected for AD (i.e., registered for AD *and* MD in the databases) and the numerator was the FGRS for cases of MD who are subject to primary ascertainment (i.e., any registration for MD in the databases). In the analyses we focus on cases of MD and AD, and in our analyses, we quantify the changes in the FGRSs for MD, AD, BD, OCD, SZ, DUD and AUD.

## Results

### Descriptive results

The descriptive statistics of our sample is provided in Table 1a for the four disorders we examine most carefully (MD, AD, DUD and AUD) ascertained from our cohort of all native Swedes born 1940–2003. Our samples for analysis for MD and AD are slightly above 800,000 and for AUD and DUD between ~225,000 and 325,000. We see the expected female excess in MD and AD and male excess and AUD and DUD. We also present the mean FGRS for these four patient cohorts. Important to the interpretation of our results is the pattern wherein the highest levels of FGRS for MD cases is found for FGRS_MD_ but that is followed closely by FGRS_AD_. In cases of AD, the highest level of FGRS_AD_ but again closely followed by FGRS_MD_. A nearly identical pattern is seen for FGRS_AUD_ and FGRS_DUD_ in cases of AUD and DUD.

**Table 1:** a Descriptive Statistics on our Main Samples: Population: Individuals born 1940–2003 in Sweden to Swedish born parents (*N* = 5,828,049). b Polychoric Correlations Matrix between the FGRSs for MD, AD, OCD, BD, SZ, DUD and AUD in the entire native population born 1945–2003^a^.

a							
	MD	AD	DUD	AUD	SZ	BD	OCD
N individuals (%)	806,320 (13.8%)	836,493 (14.4%)	225,140 (3.9%)	327,706 (5.6%)	23,419 (0.4%)	70,638 (1.2%)	41,987 (0.7%)
Year of Birth (Mean, SD)	1970 (17)	1973 (17)	1975 (16.8)	1961 (15.1)	1959 (13.3)	1969 (16.6)	1980 (15.0)
Females	63.0%	63.4%	33.4%	28.0%	38.4%	61.2%	59.5%
Mean FGRS (SD)							
MD	0.29 (1.1)^b^	0.27 (1.1)^a^	0.30 (1.1)	0.21 (1.1)	0.06 (1.0)	0.40 (1.2)^a^	0.31 (1.2)
AD	0.26 (1.1)^a^	0.31 (1.1)^b^	0.34 (1.1)	0.25 (1.1)	0.07 (1.0)	0.33 (1.1)	0.36 (1.2)^b^
DUD	0.17 (1.2)	0.20 (1.2)	0.67 (1.6)^b^	0.44 (1.4)^a^	0.18 (1.2)	0.30 (1.3)	0.14 (1.2)
AUD	0.16 (1.1)	0.19 (1.1)	0.59 (1.4)^a^	0.54 (1.4)^b^	0.20 (1.2)	0.27 (1.2)	0.13 (1.1)
SZ	0.03 (1.1)	0.05 (1.1)	0.10 (1.2)	0.06 (1.2)	0.73 (2.8)^b^	0.17 (1.5)	0.09 (1.3)
BD	0.09 (1.1)	0.10 (1.2)	0.16 (1.2)	0.11 (1.1)	0.20 (1.3)^a^	0.61 (1.9)^b^	0.12 (1.2)
OCD	0.07 (1.2)	0.09 (1.2)	0.06 ((1.1)	0.04 (1.0)	0.06 (1.1)	0.09 (1.2)	0.31 (1.6)^a^

b							
	FGRS_MD_	FGRS_AD_	FGRS_OCD_	FGRS_BD_	FGRS_SZ_	FGRS_DUD_	FGRS_AUD_
FGRS_MD_	1						
FGRS_AD_	0.56	1					
FGRS_OCD_	0.24	0.30	1				
FGRS_BD_	0.33	0.26	0.17	1			
FGRS_SZ_	0.11	0.12	0.14	0.29	1		
FGRS_DUD_	0.32	0.35	0.14	0.28	0.20	1	
FGRS_AUD_	0.27	0.29	0.11	0.22	0.17	0.52	1

^a^The FGRS are categorized into groups based on results from K-means clustering. The number of thresholds is based on the ratio “between sum of squares” and “total sum of square” (3 thresholds for all FGRSs).

^b^Highest FGRS level for each disorder.

^c^Second highest FGRS for each disorder.

We present, in Table 1b, a polychoric correlation matrix for the FGRS across seven diagnoses used in this paper. The two highest correlations were for the MD/AD (+0.56) and AUD/DUD pairs (+0.52). Four other correlations were above 0.30: DUD/AD, BD/MD, DUD/MD and OCD/AD.

### General comorbidity

We illustrate our approach to the examination of the impact of general comorbidity and our FGRS ratio statistic in Fig. 1. The upper left-hand corner shows the FGRS scores for seven disorders in our MD cohort in light grey compared to that cohort from which all cases with a comorbid AD diagnosis have been removed, shown in dark grey. As expected, the FGRS scores for all disorders in this MD cohort decline when comorbid AD cases are removed. Parallel results are presented for DUD and AUD in the upper right and lower-left panels. Note that the Y-axis differ in these figures because the FGRS scores for DUD and AUD are quite a bit higher than for MD.

**Fig. 2:**
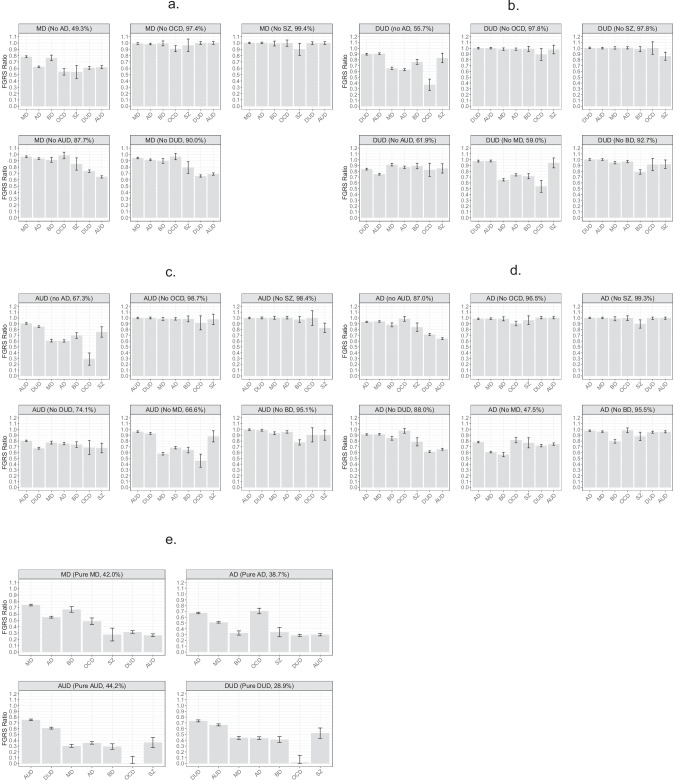
Impact of Comorbidity on Family Genetic Risk Scores Across Major Psychiatric Disorders. **a** Family genetic risk score (FGRS) Ratio Scores (with 95% CIs) for Cases of Major Depression (MD) With and Without Comorbid Cases of Anxiety Disorder (AD), OCD, Schizophrenia (SZ), Alcohol Use Disorder (AUD) and Drug Use Disorder (DUD), along with % of total MD Sample Remaining After the Elimination of the Comorbid Cases. The Y-Axis is the mean FGRS ratio which is explained in more in the methods section but is defined as the ratio of genetic risk for a particular disorder A (e.g., AUD) in a representative cohort of cases of disorder B (e.g., MD) where a particular psychiatric disorder C (e.g., DUD) has been screened out compared to the level of genetic risk for disorder A (AUD) in an unselected cohort of cases of disorder B (MD). **b** FGRS Ratio Scores (with 95% CIs) for Cases of Drug Use Disorder (DUD) With and Without Comorbid Cases of Anxiety Disorder (AD), OCD, Schizophrenia (SZ), Alcohol Use Disorder (AUD), Major Depression (MD) and Bipolar Disorder (BD), along with % of total MD Sample Remaining After the Elimination of the Comorbid Cases. The Y-Axis is the mean FGRS ratio. **c** FGRS Ratio Scores (with 95% CIs) for Cases of Alcohol Use Disorder (AUD) With and Without Comorbid Cases of Anxiety Disorder (AD), OCD, Schizophrenia (SZ), Drug Use Disorder (DUD), Major Depression (MD) and Bipolar Disorder (BD), along with % of total MD Sample Remaining After the Elimination of the Comorbid Cases. The Y-Axis is the mean FGRS ratio. **d** FGRS Ratio Scores (with 95% CIs) for Cases of Anxiety Disorder (AD) With and Without Comorbid Cases of Alcohol Use Disorder (AUD), OCD, Schizophrenia (SZ), Drug Use Disorder (DUD), Major Depression (MD) and Bipolar Disorder (BD), along with % of total MD Sample Remaining After the Elimination of the Comorbid Cases. The Y-Axis is the mean FGRS ratio. **e** FGRS Ratio Scores (with 95% CIs) for Cases of Major Depression (MD), Anxiety Disorder (AD), Alcohol Use Disorder (AUD), and Drug Use Disorder (DUD) after the elimination of cases comorbid for MD with AD, OCD, SZ, AUD and DUD, comorbid for AD with MD, OCD, SZ, AUD and DUD, comorbid for AUD with MD, AD, OCD, SZ, and DUD and comorbid for DUD with MD, AD, OCD, SZ and AUD. The Y-Axis is the mean FGRS ratio.

To quantify this decline for MD we present, in Fig. 2a, the FGRS ratio described above. From the left-hand corner of Fig. 2a, we can easily see that although the FGRS levels decline for all disorders with the removal of comorbid AD cases, these declines are not equal. They are proportionally strongest for FGRS_OCD_ and FGRS_SZ_ and weakest for FGRS_MD_ and FGRS_BD_.

Turning to an examination of the five analyses presented in Fig. 2a with our MD cohort, we observe three major patterns of changes in the FGRS ratio when specific comorbid disorders are excluded. The first pattern occurs when we eliminated comorbid cases of relatively rare disorders which occurred in under 5% of all our depressive cases: OCD and SZ. Here, the FGRS ratios were close to one for all disorders except that for the specific comorbid disorder that was eliminated from the MD cohort. The second pattern was seen when we excluded cases with the disorder with the highest prevalence in the MD cohort: AD. Here we see a broad pattern of changes across all the disorders with modest declines in the FGRS ratio for FGRS_MD_ and FGRS_BD_ to around +0.75 and more substantial drops, below +0.65, for the other five disorders. The third pattern was seen when we eliminated cases that were comorbid disorders with an intermediate prevalence in MD cases: AUD and DUD. This produced moderate reductions in the FGRS ratio for the disorder eliminated (+0.60 to +0.65), slightly less substantial reductions in the other substance use disorder and small reductions for the remaining disorders.

Fig. 2b examines the impact of censoring various comorbid disorders in our cohort of DUD cases. As with MD, eliminating comorbid cases of relatively rare co-morbid disorders – in this case OCD, BD and SZ – produced only modest declines in the FGRS ratio for the excluded disorder and a few quite modest changes for related syndromes (e.g., an FGRS ratio of 0.94 for MD when BD cases were eliminated). By contrast, eliminating all cases comorbid with MD and AD (which included 44 and 41% of all DUD cases, respectively) produced wide-spread reductions in the FGRS ratio so that a DUD cohort with all MD or all AD cases eliminated had appreciable reductions in genetic risks not only for MD but also for AD, BD and OCD. These disorders all had appreciable FGRS correlations with MD and AD which likely plays a role in these reductions. By comparison, there is little reduction in the FGRS ratio for SZ in these two examples, which has a very modest FGRS correlation with MD or AD (Table 1b). Of note, when eliminating comorbid MD cases, the FGRS ratio for genetic risk for DUD, barely declined while the elimination of comorbid AD cases dropped the FGRS ratio to 0.90. Eliminating cases comorbid with AUD (around 32% of all DUD cases) produced a unique pattern of FGRS ratios, with the FGRS ratio for AUD dropping moderately to around 0.75, and modestly, to between +0.80 and 0.90, for all other genetic risks.

We then present, in Fig. 2c, d, parallel results for our AUD and AD case cohorts. As expected, the results for AUD are similar to those found for DUD and the findings for AD resemble those seen for MD.

The final analyses of our general comorbidity question were to examine, in Fig. 2e, the effects of eliminating, *at the same time*, comorbidity to five difference disorders for MD, AD, AUD and DUD. For all four disorders, the pattern of FGRS ratios is substantially altered. For the primary disorder, the FGRS ratio is moderately reduced (0.65–0.75), but for some of the other genetic risks, the reduction is much more marked. For example, in AUD, the FGRS ratios for FGRS_MD_, FGRS_AD_, FGRS_BD_ and FGRS_OCD_ are all substantially reduced, with values below 0.35.

### Special comorbidity

The onset of certain psychiatric or substance use disorders might predispose to the development of subsequent conditions. If correct, it might, for a genetic study, be prudent to eliminate cases who had a prior onset of that predisposing disorder. Such cases would then likely have a reduced genetic risk than more typical case probands. Of all the potential predisposing conditions for psychiatric illnesses, prior substance use disorders are often suspected of playing such a potential role. Therefore, we examined, in Table 2, the mean genetic risks for our MD, BD and SZ cases who did versus did not have prior onsets, over variable time periods, of AUD and DUD.

**Table 2:** MD, SZ, BD cases where cases with AUD/DUD onset before MD, SZ, BD are eliminated over different time frames.

Sample	Prior	Mean FGRS	Anytime prior	2 years prior	1 year prior
SZ	DUD	0.731 (0.696; 0.765	0.758 (0.720; 0.796)	0.746 (0.710; 0.783)	0.744 (0.708; 0.780)
SZ	AUD		0.750 (0.712; 0.788)	0.739 (0.703; 0.75)	0.735 (0.700; 0.770)
BD	DUD	0.614 (0.600; 0.628)	0.627 (0.611; 0.642)	0.621 (0.607; 0.636)	0.619 (0.604; 0.634)
BD	AUD		0.629 (0.614; 0.645)	0.626 (0.611; 0.641)	0.623 (0.609; 0.638)
MD	DUD	0.290 (0.288; 0.293)	0.284 (0.281; 0.286)	0.287 (0.284; 0.289)	0.287 (0.285; 0.290)
MD	AUD		0.286 (0.283; 0.288)	0.289 (0.286; 0.291)	0.289 (0.287; 0.292)

All cases of SZ in our cohort had a mean FGRS_SZ_ of 0.731. If we eliminate from this patient cohort, individuals with a diagnosis of DUD any time prior to the SZ onset, or just within 2 years or within only 1 year of SZ onset, the FGRS increases only modestly and non-significantly to, respectively, 0.758, 0. 746 and 0.744. Results are very similar for eliminating, in our SZ cohort, cases with prior AUD. The same pattern of findings is seen with BD – with only small non-significant increases seen in their FGRS_BD_ with the elimination of cases with prior DUD or AUD. All cases of MD in our cohort had a mean FGRS_MD_ of 0.290. When we examined the FGRS_MD_ in this cohort when cases of DUD or AUD were been eliminated across our three different time periods, no appreciable change was seen.

### Secondary ascertainment

**Fig. 3:**
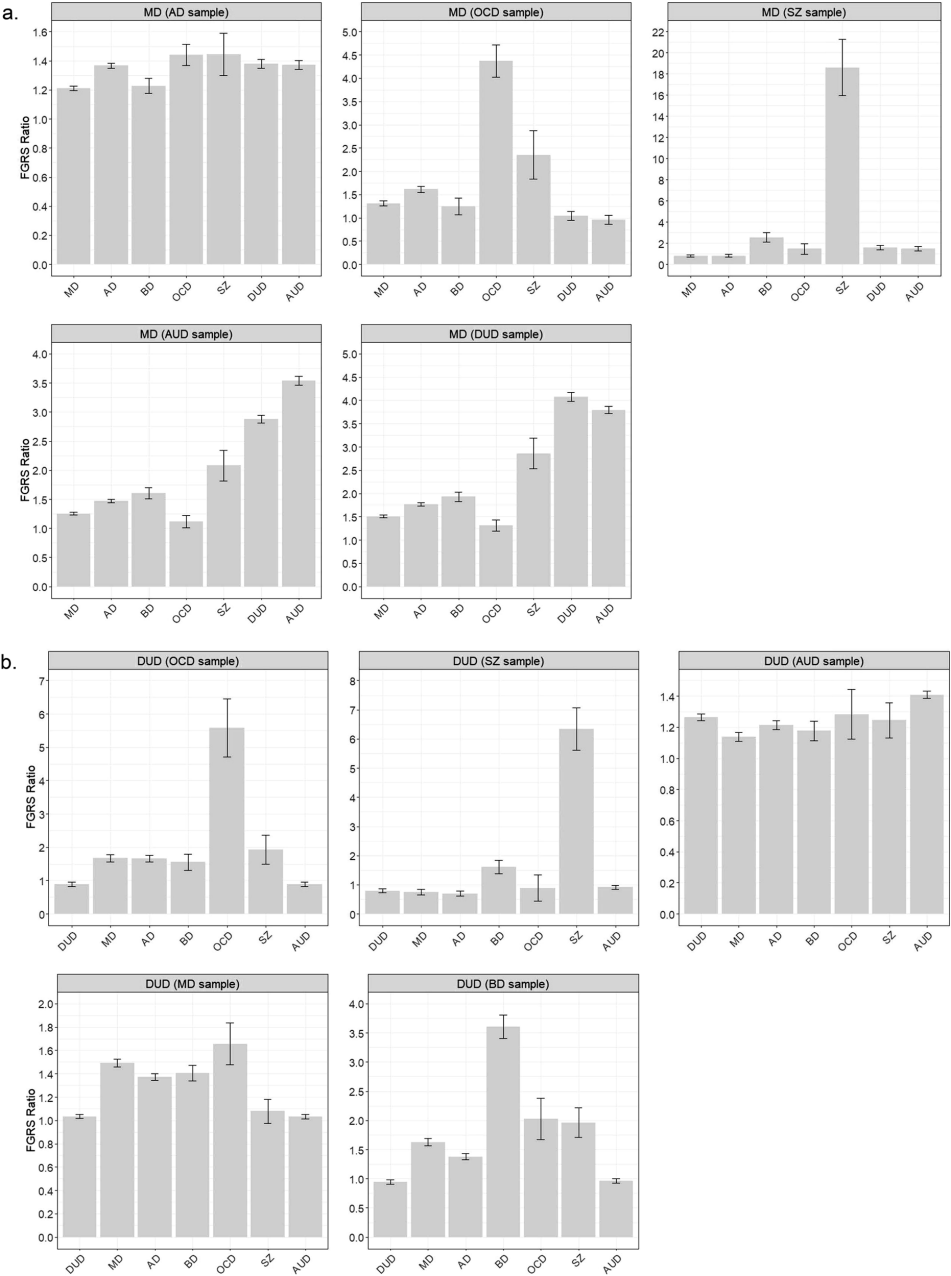
Comparison of Family Genetic Risk Scores by Primary vs. Secondary Case Ascertainment. **a** FGRS Ratio Scores (with 95% CIs) of cases of MD who are subject to primary ascertainment versus secondary ascertainment of cases of MD in samples already collected for, respectively, AD, OCD, SZ, AUD and DUD. The Y-Axis is the mean FGRS ratio. **b** FGRS Ratio Scores (with 95% CIs) of cases of DUD who are subject to primary ascertainment versus secondary ascertainment of cases of DUD in samples already collected for, respectively, OCD, SZ, AUD, MD and BD. The Y-Axis is the mean FGRS ratio.

We began by examining, in Fig. 3a, the impact of selecting cases of MD from patient cohorts primarily ascertained for having AD, OCD, SZ, AUD and DUD. Here, the FGRS ratios reflected the change in levels of genetic risk for various disorder in cases of primarily ascertained MD versus those secondarily ascertained through these other disorders. We see three different profiles. First, for cases of MD ascertained through the rare disorders like OCD and SZ, we see large dramatic increase in the FGRS ratio for that specific disorder and less substantial increases for disorders that are substantially genetic correlated with them (e.g., BD for SZ and AD for OCD). Second, for MD cases ascertained through AUD or DUD, we see large increases in the FGRS ratio for those substance use disorders but also for SZ and, more modestly, BD. Third, cases of MD ascertained through AD show a distinct profile with modest increases in the FGRS ratios of 1.2–1.4 across all disorders.

In Fig. 3b, we explored the patterns of FGRS ratios for cases of DUD secondarily ascertained from samples of cases with OCD, SZ, AUD, MD and BD. As with MD in Fig. 3a, for the rarer disorders of OCD, SZ and BD, the very large increase in the FGRS ratio was mostly restricted to those disorders, although for BD, the increase “bled” into the related disorders of OCD, MD and SZ. For DUD cases ascertained through MD samples, we see moderate rises in the FGRS ratio for MD and 3 associated disorders of AD, BD and OCD. Finally, in cases of DUD ascertained from a sample of AUD cases demonstrates a modest elevation of the FGRS ratio across all the disorders examined.

## Discussion

We here sought to examine, in Swedish national samples, the impact of changes in case ascertainment on the patterns of observed genetic risks as assessed through our FGRS. We sought to address three questions commonly discussed in current psychiatric GWAS studies: (i) what to do with patterns of case comorbidity? (ii) whether the genetic signal from a case cohort could be increased by excluding cases that might be secondary to another predisposing disorder? and (iii) what changes in genetic architecture of a case sample arises if secondary ascertainment is employed, that is finding samples of disorder A in cohorts originally ascertained for disorder B? We discuss our results and the implications for each of these issues in turn, noting that our goal was to be illustrative of possible patterns of findings rather than to be exhaustive in exploring all possible comorbid combinations of disorders.

To address the first question, we examined the impact of eliminating cases comorbid for six disorders from each of our four primary conditions: MD, AD, DUD and AUD. We found that the effects of such selection depended on three factors: (i) the prevalence of the excluded disorder, (ii) the level of shared genetic risk of the excluded comorbid disorder with the primary disorder and (iii) the pattern of sharing of genetic risks of the excluded disorder with other comorbid disorders. Rare disorders with only modest genetic correlations with the primary or other comorbid disorders of interest had relatively specific effects, reducing the genetic risk in the cases only for the excluded disorder. By contrast, censoring common comorbid disorders that were at least moderately genetically correlated with the primary disorder tended to have broad effects, decreasing genetic risk for not only the primary disorder but a range of other comorbid disorders in the case cohort. We see this most robustly when eliminating comorbid AD cases in the MD cohort and AUD cases in a DUD cohort. This finding makes sense in that MD and AD share an appreciable proportion of their genetic risk genes and therefore comorbid cases would be expected to have higher average genetic risk for both disorders compared to a case with only MD or only AD. Furthermore, if a researcher eliminated comorbid cases not closely genetically related to the primary disorder, but to other secondary disorders, that would reduce the FGRS ratio not only for that disorder but for those closely related to it. This is seen in censoring cases of MD from a DUD cohort. The level of the genetic risk for DUD is not changed much, but a reduction is seen in the genetic risk for not only MD, but also disorders which share genetic risk to MD: AD, BD and OCD.

To address our second question, we examined whether the level of the genetic risk for three psychiatric disorders – SZ, BD and MD – was increased if we screened out affected individuals whose onset of the main disorder was preceded by the prior onset of DUD or AUD over a range of time periods. Our results were uniformly negative. While few doubt that all of these disorders are etiologically heterogenous and some of those subforms might be less genetically influenced than others, using prior diagnoses of substance use disorders in national Swedish data was not an effective means of identifying, and thereby screening out, potential “phenocopies.”

Our third question involved studying the effect of secondary ascertainment, where disorder A is ascertained as a comorbid diagnosis in a sample collected for disorder B. We found that it could, in certain situations, produces large increases in the genetic risk for disorder B and associated disorders in the cohort of patients with disorder A. This could have a range of downstream consequences, in particular biasing upward, perhaps substantially, genetic correlations between disorder A and B. However, if disorder B is closely genetically related to disorder A (as seen with MD/AD and DUD/AUD pairings), the pattern differs and produces a general and moderate elevation across the genetic risk profile. As noted above, this is because comorbid cases of closely related disorders will tend to have a higher genetic risk to both disorders than cohorts with just one or the other.

### Limitations

These results should be interpreted in the context of four potent limitations. First, diagnoses obtained from high quality Swedish national registries will not replicate findings based on personal interviews. However, registry diagnoses have a number of advantages including lack of cooperation or recall bias. Efforts to study the validity of psychiatric diagnoses in the Swedish registries have generally been reassuring [23–31]. Second, the FGRS, a family phenotype-based measure to assess quantitative genetic risk distinct from polygenic risk scores, has been now widely published [16–18, 32–35], with evidence that it is not highly sensitive to assumptions involved in its calculation, that the correction for cohabitation effects performs appropriately, and the method agrees well with other similar statistical approaches [36, 37]. Furthermore, empirical and simulations analyses suggest that the observed modest correlations between FRGS-like statistics and PRS from the Danish iPsych study are consistent with the hypothesis that phenotype-based extended family measures like the FGRS, and molecular polygene scores are both fallible measures of the same underlying large set of small effect genetic risk alleles [37–39]. Third, we cannot be certain of the degree to which our results generalize beyond the Swedish population. This would predict that the broad pattern of findings reported here from Sweden using FGRS would likely be replicated in parallel analyses of PRS in other samples. Fourth, our point here was to alert researchers to the potential biases or successes of certain ascertainment strategies, rather than presented a framework in which to “correct” results from such biases.

## Conclusions

We would emphasize three general results from these analyses. First, the elimination of comorbid conditions from case cohorts can have substantial impacts on the pattern of genetic risks for the remaining cases both of the primary disorder and for a range of other disorders. But the magnitude and significance of these changes varies as a function of the frequency of the comorbid disorder and its genetic relationship to the primary disorder and other possible comorbid conditions. Caution should therefore be used in applying such screening methods in molecular genetic studies. Second, with respect to prior substance use disorders, our attempt to increase the genetic risk of case cohorts by eliminating potential “secondary” cases was unsuccessful. Third, secondary ascertainment can have very large effects on the pattern of genetic risks for cases and suggests that this method should be sparingly used if at all in future studies. Careful attention to case selection strategies in GWAS studies will likely pay-off in the generalizability and quality of downstream results.

### Data analysis

Kristina Sundquist MD PhD had full access to all the data in the study and takes responsibility for the integrity of the data and the accuracy of the data analysis. Anyone wishing to implement the FGRS in their own data can contact Dr. Ohlsson at Henrik.Ohlsson@med.lu.se for assistance.

## Supplementary information

Supplement to Exploring the Implications of Case Selection Methods for Psychiatric Molecular Genetic Studies

## Data Availability

The data for this study are not publicly available due to legal restrictions with regard to the nationwide Swedish registers, but they can be acquired directly from the responsible authorities pending their approval.

## References

[1] Kendler KS, Chatzinakos C, Bacanu SA. The impact on estimations of genetic correlations by the use of super-normal, unscreened, and family-history screened controls in genome wide case-control studies. Genet Epidemiol. 2020;44:283–9. 31961015 10.1002/gepi.22281

[2] Benstock SE, Weaver K, Hettema JM, Verhulst B. Using alternative definitions of controls to increase statistical power in GWAS. Behav Genet. 2024:1–14. 10.21203/rs.3.rs-3858178/v1. 10.1007/s10519-024-10187-w PMC11661655 38869698

[3] Kessler RC. The prevalence of psychiatric comorbidity. In: Wetzler S, Sanderson WC, editors. Treatment strategies for patients with psychiatric comorbidity. New York, NY: John Wiley & Sons, Inc.; 1997. p. 23–48.

[4] Kessler RC, Chiu WT, Demler O, Merikangas KR, Walters EE. Prevalence, severity, and comorbidity of 12-month DSM-IV disorders in the National Comorbidity Survey Replication. Arch Gen Psychiatry. 2005;62:617–27. 15939839 10.1001/archpsyc.62.6.617 PMC2847357

[5] Compton WM, Thomas YF, Stinson FS, Grant BF. Prevalence, correlates, disability, and comorbidity of DSM-IV drug abuse and dependence in the United States: results from the national epidemiologic survey on alcohol and related conditions. Arch Gen Psychiatry. 2007;64:566–76. 17485608 10.1001/archpsyc.64.5.566

[6] Regier DA, Farmer ME, Rae DS, Locke BZ, Keith SJ, Judd LL, et al. Comorbidity of mental disorders with alcohol and other drug abuse. Results from the Epidemiologic Catchment Area (ECA) Study. JAMA. 1990;264:2511–8. 2232018

[7] Merikangas KR, Angst J, Eaton W, Canino G, Rubio-Stipec M, Wacker H, et al. Comorbidity and boundaries of affective disorders with anxiety disorders and substance misuse: results of an international task force. 8864150

[8] Oldham JM, Skodol AE, Kellman HD, Hyler SE, Doidge N, Rosnick L, et al. Comorbidity of axis I and axis II disorders. Am J Psychiatry. 1995;152:571–8. 7694906 10.1176/ajp.152.4.571

[9] Crebbin K, Mitford E, Paxton R, Turkington D. First-episode drug-induced psychosis: a medium term follow up study reveals a high-risk group. Soc Psychiatry Psychiatr Epidemiol. 2009;44:710–5. 19183816 10.1007/s00127-008-0490-2

[10] Niemi-Pynttari JA, Sund R, Putkonen H, Vorma H, Wahlbeck K, Pirkola SP. Substance-induced psychoses converting into schizophrenia: a register-based study of 18,478 Finnish inpatient cases. J Clin Psychiatry. 2013;74:e94–e9. 23419236 10.4088/JCP.12m07822

[11] Kendler KS, Ohlsson H, Sundquist J, Sundquist K. Prediction of onset of substance-induced psychotic disorder and its progression to schizophrenia in a Swedish national sample. Am J Psychiatry. 2019;176:711–9. 31055966 10.1176/appi.ajp.2019.18101217 PMC6718312

[12] Arendt M, Rosenberg R, Foldager L, Perto G, Munk-Jorgensen P. Cannabis-induced psychosis and subsequent schizophrenia-spectrum disorders: follow-up study of 535 incident cases. Br J Psychiatry. 2005;187:510–5. 16319402 10.1192/bjp.187.6.510

[13] Gelernter J, Kranzler HR, Sherva R, Koesterer R, Almasy L, Zhao H, et al. Genome-wide association study of opioid dependence: multiple associations mapped to calcium and potassium pathways. Biol Psychiatry. 2014;76:66–74. 24143882 10.1016/j.biopsych.2013.08.034 PMC3992201

[14] Gaddis N, Mathur R, Marks J, Zhou L, Quach B, Waldrop A, et al. Multi-trait genome-wide association study of opioid addiction: OPRM1 and beyond. Sci Rep. 2022;12:16873. 36207451 10.1038/s41598-022-21003-y PMC9546890

[15] Strom NI, Verhulst B, Bacanu S-A, Cheesman R, Purves KL, Gedik H, et al. Genome-wide association study of major anxiety disorders in 122,341 European-ancestry cases identifies 58 loci and highlights GABAergic signaling. medRxiv [Preprint] 2024; e-pub ahead of print; 10.1101/2024.07.03.24309466; https://www.medrxiv.org/content/10.1101/2024.07.03.24309466v1 (accessed 3 September 2024). 10.1038/s41588-025-02485-8 PMC12900644 41634414

[16] Kendler KS, Ohlsson H, Sundquist J, Sundquist K. Family genetic risk scores and the genetic architecture of major affective and psychotic disorders in a Swedish national sample. JAMA Psychiatry. 2021;78:735–43. 33881469 10.1001/jamapsychiatry.2021.0336 PMC8060884

[17] Kendler KS, Ohlsson H, Bacanu S, Sundquist J, Sundquist K. Differences in genetic risk score profiles for drug use disorder, major depression, and ADHD as a function of sex, age at onset, recurrence, mode of ascertainment, and treatment. Psychol Med. 2023;53:3448–60. 35098912 10.1017/S0033291721005535 PMC10863503

[18] Kendler KS, Ohlsson H, Moscicki EK, Sundquist J, Edwards AC, Sundquist K. Genetic liability to suicide attempt, suicide death, and psychiatric and substance use disorders on the risk for suicide attempt and suicide death: a Swedish national study. Psychol Med. 2023;53:1639–48. 37010214 10.1017/S0033291721003354 PMC10916711

[19] Kendler KS, Ohlsson H, Sundquist J, Sundquist K. Impact of comorbidity on family genetic risk profiles for psychiatric and substance use disorders: a descriptive analysis. Psychol Med. 2023;53:2389–98. 37310304 10.1017/S0033291721004268 PMC10832607

[20] Kendler KS, Ohlsson H, Sundquist J, Sundquist K. The relationship between familial-genetic risk and pharmacological treatment in a Swedish national sample of patients with major depression, bipolar disorder, and schizophrenia. Mol Psychiatry. 2024;29:742–9. 38123723 10.1038/s41380-023-02365-9

[21] Lee SH, Ripke S, Neale BM, Faraone SV, Purcell SM, Perlis RH, et al. Genetic relationship between five psychiatric disorders estimated from genome-wide SNPs. Nat Genet. 2013;45:984–94. 23933821 10.1038/ng.2711 PMC3800159

[22] Grotzinger AD, Mallard TT, Akingbuwa WA, Ip HF, Adams MJ, Lewis CM, et al. Genetic architecture of 11 major psychiatric disorders at biobehavioral, functional genomic and molecular genetic levels of analysis. Nat Genet. 2022;54:548–59. 35513722 10.1038/s41588-022-01057-4 PMC9117465

[23] Lichtenstein P, Bjork C, Hultman CM, Scolnick E, Sklar P, Sullivan PF. Recurrence risks for schizophrenia in a Swedish national cohort. Psychol Med. 2006;36:1417–25. 16863597 10.1017/S0033291706008385

[24] Sellgren C, Landen M, Lichtenstein P, Hultman CM, Langstrom N. Validity of bipolar disorder hospital discharge diagnoses: file review and multiple register linkage in Sweden. Acta Psychiatr Scand. 2011;124:447–53. 21838734 10.1111/j.1600-0447.2011.01747.x

[25] Ekholm B, Ekholm A, Adolfsson R, Vares M, Osby U, Sedvall GC, et al. Evaluation of diagnostic procedures in Swedish patients with schizophrenia and related psychoses. Nord J Psychiatry. 2005;59:457–64. 16316898 10.1080/08039480500360906

[26] Johansson V, Hultman CM, Kizling I, Martinsson L, Borg J, Hedman A, et al. The schizophrenia and bipolar twin study in Sweden (STAR). Schizophr Res. 2019;204:183–92. 30121189 10.1016/j.schres.2018.08.001 PMC6377356

[27] Kendler KS, Ohlsson H, Lichtenstein P, Sundquist J, Sundquist K. The genetic epidemiology of treated major depression in Sweden. Am J Psychiatry. 2018;175:1137–44. 30021458 10.1176/appi.ajp.2018.17111251

[28] Sundquist J, Ohlsson H, Sundquist K, Kendler KS. Common adult psychiatric disorders in Swedish primary care (Where most mental health patients are treated). BMC Psychiatry. 2017;17:235. 28666429 10.1186/s12888-017-1381-4 PMC5493066

[29] Kendler KS, Lonn SL, Salvatore J, Sundquist J, Sundquist K. The origin of spousal resemblance for alcohol use disorder. JAMA Psychiatry. 2018;75:280–6. 29417130 10.1001/jamapsychiatry.2017.4457 PMC5885945

[30] Kendler KS, Ji J, Edwards AC, Ohlsson H, Sundquist J, Sundquist K. An extended Swedish national adoption study of alcohol use disorder. JAMA Psychiatry. 2015;72:211–8. 25565339 10.1001/jamapsychiatry.2014.2138 PMC4351126

[31] Ludvigsson JF, Andersson E, Ekbom A, Feychting M, Kim JL, Reuterwall C, et al. External review and validation of the Swedish national inpatient register. BMC Public Health. 2011;11:450. 21658213 10.1186/1471-2458-11-450 PMC3142234

[32] Kendler KS, Ohlsson H, Sundquist J, Sundquist K. The patterns of family genetic risk scores for eleven major psychiatric and substance use disorders in a Swedish national sample. Transl Psychiatry. 2021;11:326. 34045441 10.1038/s41398-021-01454-z PMC8160183

[33] Kendler KS, Ohlsson H, Sundquist J, Sundquist K. The impact of sex, age at onset, recurrence, mode of ascertainment and medical complications on the family genetic risk score profiles for alcohol use disorder. Psychol Med. 2023;53:1732–40. 34620257 10.1017/S0033291721003317

[34] Kendler KS, Ohlsson H, Sundquist J, Sundquist K. The moderation of the genetic risk for alcohol and drug use disorders in a Swedish national sample by the genetic aptitude for educational attainment. Psychol Med. 2023;53:3077–84. 37449484 10.1017/S0033291721005134 PMC10953683

[35] Kendler KS, Rosmalen JGM, Ohlsson H, Sundquist J, Sundquist K. A distinctive profile of family genetic risk scores in a Swedish national sample of cases of fibromyalgia, irritable bowel syndrome, and chronic fatigue syndrome compared to rheumatoid arthritis and major depression. Psychol Med. 2023;53:3879–86. 35354508 10.1017/S0033291722000526 PMC10317803

[36] Hujoel ML, Gazal S, Loh P-R, Patterson N, Price AL. Liability threshold modeling of case–control status and family history of disease increases association power. Nat Genet. 2020;52:541–7. 32313248 10.1038/s41588-020-0613-6 PMC7210076

[37] Dybdahl Krebs M, Appadurai V, Georgii Hellberg K-L, Ohlsson H, Steinbach J, Pedersen E, et al. The relationship between genotype- and phenotype-based estimates of genetic liability to human psychiatric disorders, in practice and in theory. medRxiv [Preprint] 2023; e-pub ahead of print 2023; 10.1101/2023.06.19.23291606v3 (accessed 5 September 2024). 10.1016/j.ajhg.2025.11.016 PMC12824625 41435841

[38] Dybdahl Krebs M, Georgii Hellberg K-L, Lundberg M, Appadurai V, Ohlsson H, Pedersen E, et al. PA-FGRS is a novel estimator of pedigree-based genetic liability that complements genotype-based inferences into the genetic architecture of major depressive disorder. medRxiv [Preprint] 2023; e-pub ahead of print; 10.1101/2023.06.23.23291611; https://www.medrxiv.org/content/10.1101/2023.06.23.23291611v2 (accessed 7 September 2024).

[39] Dybdahl Krebs M, Georgii Hellberg KL, Lundberg M, Appadurai V, Ohlsson H, Pedersen E, et al. Genetic liability estimated from large-scale family data improves genetic prediction, risk score profiling, and gene mapping for major depression. Am J Hum Genet. 2024;111:2494–509. 39471805 10.1016/j.ajhg.2024.09.009 PMC11568754

