# Supplementary material for: Exploring the implications of case selection methods for psychiatric molecular genetic studies
Source: Mol Psychiatry. 2025 Apr 20;30(9):4334–42. doi: 10.1038/s41380-025-03015-y (PMC12339360; doi:10.1038/s41380-025-03015-y)
Supplement: Supplementary file 1 — Supplement to Exploring the Implications of Case Selection Methods for Psychiatric Molecular Genetic Studies [file 41380_2025_3015_MOESM1_ESM.docx]

Appendix

**Table 1 - Description of Registers**

*National Patient Register*

In the 1960's the National Board of Health and Welfare started to collect information regarding in-patients at public hospitals, the National Patient Register (NPR). Initially it contained information about all patients treated in psychiatric care and approximately 16 percent of patients in somatic care. The register at that time covered six of the 26 county councils in Sweden. In 1984, the Ministry of Health and Welfare together with the Federation of County Councils decided a mandatory participation for all county councils. From 1987, NPR includes all in-patient care in Sweden. Since 2001, the register also covers outpatient doctor visits including day surgery and psychiatric care from both private and public caregivers. For more information, see https://www.socialstyrelsen.se/en/statistics-and-data/registers/register-information/the-national-patient-register/

*Primary Care Data*

We also used information from Primary Care. This is a research dataset including individual-level information on clinical diagnoses from primary health care centers. In the end of the follow-up period the registers covers almost 100% of the population. The figure below show the percentage of the entire Swedish population that resides in counties with primary care data. For more information see: Sundquist, J., Ohlsson, H., Sundquist, K., Kendler, KS. Common adult psychiatric disorders in Swedish primary care where most mental health patients are treated. BMC Psychiatry 17, 235 (2017). https://doi.org/10.1186/s12888-017-1381-4

*
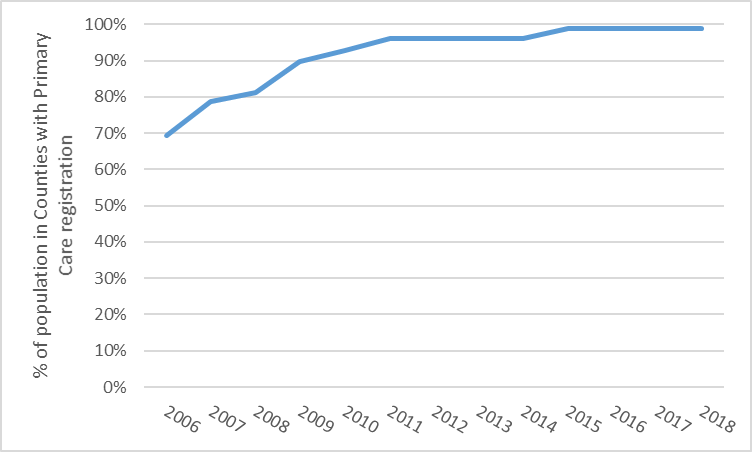
*

**Table 2 - Definition of Variables**

|  | Registers Used | Definition |
| --- | --- | --- |
| Major Depression (MD) | The National Patient Register, Primary Care data | ICD-8: 296.2, 298.0, 300.4; ICD-9: 296.2, 296.4, 298.0, 300.4; ICD-10: F32, F33. *Note: all cases of BD are excluded in the selection of MD cases |
| Anxiety Disorder (AD) | The National Patient Register, Primary Care data | ICD-8: 300.0, 300.2 ; ICD-9: 300A, 300C; ICD-10: F40, F41 |
| Obsessive-Compulsive Disorder [OCD] | The National Patient Register, Primary Care data | ICD-9: 300D; ICD-10: F42 |
| Bipolar Disorder (BD) | The National Patient Register, Primary Care data | ICD-8: 296.1, 296.3, 296.8, 296.9, 298.1; ICD-9: 296A, 296C, 296D, 296E, 296W, 298B; ICD-10: F30, F31*Note: a hierarchy of diagnoses between BD and SZ is used for selection of BD cases (see below) |
| Schizophrenia (SZ) | The National Patient Register, Primary Care data | ICD-8: 295.1, 295.2, 2953, 295.9, 295.6; ICD-9: 295B, 295C, 295D, 295G, 295X; ICD-10: F200, F201, F202, F203, F205, F209*Note: a hierarchy of diagnoses between BD and SZ are used is used for selection of BD cases (see below) |
| Alcohol Use Disorder (AUD) | The National Patient Register, Primary Care data, the Swedish Drug Register (2005-2018); the Swedish Mortality Register, and the Swedish Criminal Register (1973-2018) and the Swedish Suspicion Register (1998-2018) | Alcohol Use Disorder (AUD) was identified in the Swedish medical and mortality registries by ICD codes: ICD9: V79B, 305A, 357F, 571A-D, 425F, 535D, 291, 303, 980; ICD 10: E244, G312, G621, G721, I426, K292, K70, K852, K860, O354, T51, F10); in the Swedish Criminal Register and the Swedish Suspicion Register with at least two registrations of drunk driving (suspicion code 3005, law 1951:649 (paragraph 4 and 4A)) or drunk in charge of a maritime vessel (suspicion code 3201, law 1994:1009 (chapter 20, paragraph 4 and 5)); in the Prescribed Drug Register by the drugs disulfiram (Anatomical Therapeutic Chemical (ATC) Classification System N07BB01), acamprosate (N07BB03), and naltrexone (N07BB04). |
| Drug Use Disorder (DUD)) | The National Patient Register, Primary Care data, the Swedish Drug Register (2005-2018); the Swedish Mortality Register, and the Swedish Criminal Register (1973-2018) and the Swedish Suspicion Register (1998-2018) | Drug abuse (DA) was identified in the Swedish medical and mortality registries by ICD codes (ICD8: Drug dependence (304); ICD9: Drug psychoses (292) and Drug dependence (304); ICD10: Mental and behavioral disorders due to psychoactive substance use (F10-F19), except those due to alcohol (F10) or tobacco (F17)); in the Suspicion Register by codes 3070, 5010, 5011, and 5012, that reflect crimes related to DA; and in the Crime Register by references to laws covering narcotics (law 1968:64, paragraph 1, point 6) and drug-related driving offences (law 1951:649, paragraph 4, subsection 2 and paragraph 4A, subsection 2). DA was identified in individuals (excluding those suffering from cancer) in the Prescribed Drug Register who had retrieved (in average) more than four defined daily doses a day for 12 months from either of Hypnotics and Sedatives (Anatomical Therapeutic Chemical (ATC) Classification System N05C and N05BA) or Opioids (ATC: N02A). |
|  |  |  |

Table 3 - Hierarchy for individuals with a registration of Schizophrenia and Bipolar Disorder

The table below illustrates how individuals were categorized when they had at least one registration for BD and at least one registration for SZ.

|  |  | Number of lifetime SZ diagnoses in the registers | | | | |
| --- | --- | --- | --- | --- | --- | --- |
|  |  | 1 (Group 1) | 2 (Group 2) | 3-5 (Group 3) | 6-10 (Group 4) | More than 10 (Group 5) |
| Number of lifetime BD diagnoses in the registers | 1 (Group 1) | Last diagnosis | Last diagnosis | Most common diagnosis | Most common diagnosis | Most common diagnosis |
|  | 2 (Group 2) | Last diagnosis | Majority of last 3 diagnoses | Majority of last 3 diagnoses | Most common diagnosis | Most common diagnosis |
|  | 3-5 (Group 3) | Most common diagnosis | Majority of last 3 diagnoses | Majority of last 3 diagnoses | Majority of last 3 diagnoses | Majority of last 5 diagnoses |
|  | 6-10 (Group 4) | Most common diagnosis | Most common diagnosis | Majority of last 3 diagnoses | Majority of last 5 diagnoses | Majority of last 5 diagnoses |
|  | More than 10 (Group 5) | Most common diagnosis | Most common diagnosis | Majority of last 5 diagnoses | Majority of last 5 diagnoses | Majority of last 5 diagnoses |

**Table 4 - Calculation of the Familial Genetic Risk Score (FGRS)**

|  |
| --- |
| The dataset for the calculations includes:  Column1 = Identification number of the proband (Born 1945-2003)  Column2 = Identification number of the relative (1st to 5th degree relatives)  Column3 = Proportion of shared additive genetic effects (0.03125 to 0.50) with the proband  Column4 = Year of Birth of relative  Column5 = Sex of relative  Column6 = Age at registration for trait  Column7 = Age at end of follow-up (2018-12-31 or age at death, or age at emigration whichever came first) |
| Step 1: Using all unique relatives with a registration for the disorder, we non-parametrically estimated the distribution of Age at first registration. The empirical distribution is used to obtain weights for relatives without a registration for the disorder, in order to account for the proportion of the time-at-risk period they had completed at the end of follow-up. For example, for relatives at age x at end of follow-up, the weight corresponds to the proportion of relatives registered for the trait that had been registration at age x. For relatives born prior to 1958 we subtracted age at the end of follow-up with the following formula: 1958 - Year of birth of relative. This modification was done in order to control for registration effects (i.e, most registers in Sweden start in 1973 suggesting that relatives from early birth cohorts do not have the possibility to be registered at younger ages). Note that all relatives with the disorder are weighted one. |
| Step 2: Transform the binary variable (trait yes/no) into a z-score based on the threshold for each trait. The underlying liability of the individual is not assessable. Instead we estimated the mean of the underlying liability to obtain sex and birth decade specific Z-scores for relatives with the trait registration and relatives without the trait. We generate n random numbers from a N(0, 1) distribution and estimate the mean for relatives registered with the disorder (i.e., mean of the observations above the threshold) and for relatives without a registration (i.e., mean of all observation below the threshold). The thresholds are calculated for each decade of birth and sex. |
| Step 3: Correct for cohabitation effects. To estimate the cohabitation effect (i.e. “shared environment”), we created a database with all individuals in the Swedish population born in Sweden 1955-1990. We also included the number of years, during ages 0-15, that individuals resided in the same household as their biological father. We thereby were able to define two kinds of families i) “not-lived-with” father families (offspring never resided for more than 1 year in the same household or in the same community as their biological father); ii) “lived-with” father (offspring resided a minimum of 13 year in the same household as their biological father. We performed a logistic regression model with the binary trait in offspring as outcome and the binary trait in father, type of father, and their interaction as predictors. We used the interaction term as the difference of effect between genes only and genes + environment. The same approach was performed for half-siblings where we compared those who were reared together versus reared apart. The following interaction terms were used in the calculations for each of our disorders:   \|  \| Parent/Children \| Siblings \| \| --- \| --- \| --- \| \| MD \| 0.90 \| 0.89 \| \| AD \| 0.87 \| 0.81 \| \| BD \| 0.67 \| 0.77 \| \| SZ \| 0.93 \| 0.84 \| \| OCD \| 0.79 \| 0.74 \| \| DUD \| 0.92 \| 0.52 \| \| AUD \| 0.99 \| 0.69 \| |
| Step 4: Calculate the product for each relative using the four components:   1. Z-score (reflecting sex and year of birth adjusted rates) 2. Weight (reflecting the proportion of risk period they had completed) 3. Cohabitation effects 4. Proportion of shared genetic effects (0.03125 – 0.5) with the proband |
| Step 5: Average the product calculated in step 4 across all relatives to a proband |
| Step 6: Correct for the number of relatives. We multiplied the results from step 5 with a shrinkage factor. Shrinkage factor (SF): B/(B+A/C). It produces more shrinkage if B and C are small and A is large.   1. the variance of the z-score of the disorder across all relatives, 2. the variance in the mean z-score across all probands, 3. the weighted number of relatives for each proband (sum of Column 3 across each proband). |
| Step 7: Correct for difference by year of birth and county differences. There are 21 counties in Sweden. For each proband we used the county they had resided in during the maximum number of years (measured from 1969 and onwards) We standardized the risk score by year of birth and county of the proband into a z-score with mean 0 and SD 1. |

Table 5

Many standard textbooks discuss k-means in multidimensional contexts, but the concepts apply to one-dimensional data with minor adjustments. K-means is an adaptable algorithm that can be applied to data with any number of dimensions, including one-dimensional data. With one variable, k-means clustering essentially tries to partition the data into k contiguous segments along a line, where each segment corresponds to a cluster. Each segment is centered around a "centroid" that represents the mean of the points in that segment. Each cluster aims to minimize the distance between its points and the cluster's centroid (mean of the segment).

This is the r-code used:

set.seed(123)

wss<- NULL

for (i in 1:10){

fit = kmeans(dat1,centers = i, nstart = 25, algorithm="Lloyd", iter.max = 400)

wss = c(wss, fit$tot.withinss)

**The within-cluster sum squared (**the sum of squared distance between each point and its closest centroid) for different number of clusters (1 to 10). A 4-cluster solution seem to be best for all traits as seen in figure 1

Figure 1 - The within-cluster sum squared (the sum of squared distance between each point and its closest centroid) for different number of clusters (1 to 10).


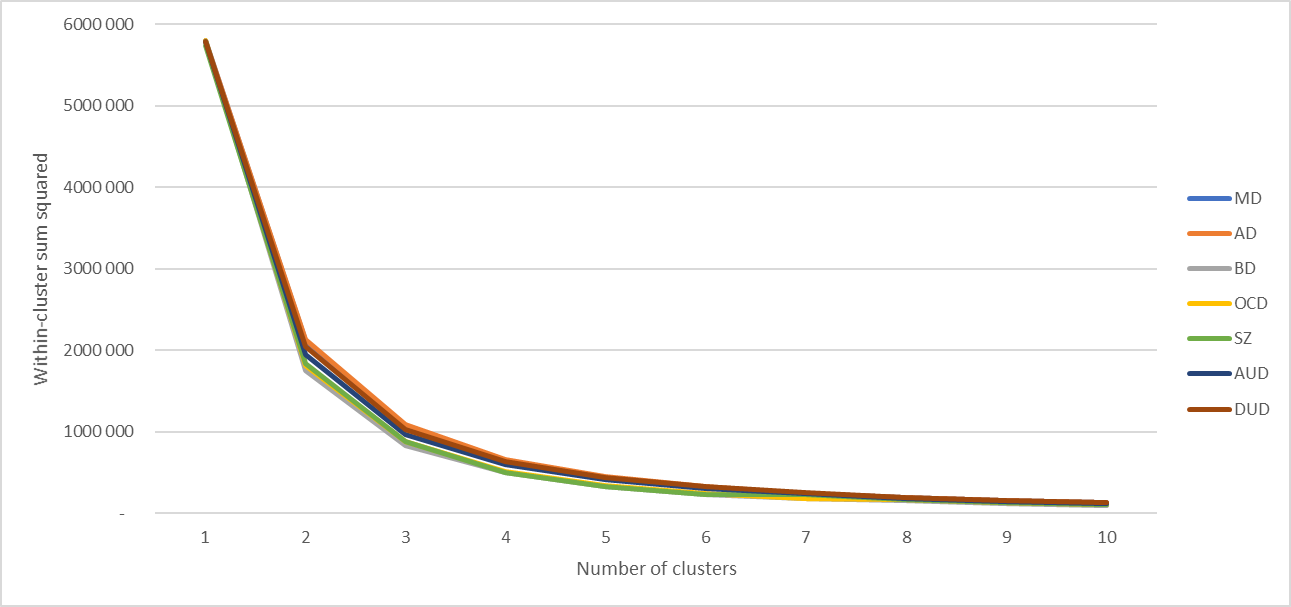


Table 6 – Polychoric Correlation

Polychoric correlation is a statistical technique used to estimate the correlation between two theoretically continuous latent variables when their observed counterparts are ordinal. The key assumptions for polychoric correlation are

Threshold Model: Each ordinal variable is assumed to represent a discretized form of a continuous latent variable. The observed ordinal categories are thought to arise from thresholds applied to the continuous latent variables.

Ordinality of Data: The observed data should be ordinal, meaning that the values represent ordered categories without precise numerical differences between them.

Sufficient Sample Size: Polychoric correlation estimates rely on large samples to provide stable and accurate results.

If these assumptions are met, the polychoric correlation can provide a more accurate estimate of the relationship between the underlying latent variables than Pearson or Spearman correlations, especially when working with ordinal data.
